# Role and Clinical Significance of Monocarboxylate Transporter 8 (MCT8) During Pregnancy

**DOI:** 10.1007/s43032-022-01162-z

**Published:** 2023-01-03

**Authors:** Jinsu Thomas, Sairoz, Anmi Jose, Vidyashree G. Poojari, Sahana Shetty, Shama Prasada K, Krishnananda Prabhu R V, Mahadev Rao

**Affiliations:** 1 Department of Pharmacy Practice, Center for Translational Research, Manipal College of Pharmaceutical Sciences, Manipal Academy of Higher Education, Manipal, 576104 Karnataka India; 2 Department of Biochemistry, Kasturba Medical College, Manipal, Manipal Academy of Higher Education, Manipal, 576104 Karnataka India; 3 Department of Reproductive Medicine and Surgery, Kasturba Medical College, Manipal, Manipal Academy of Higher Education, Manipal, 576104 Karnataka India; 4 Department of Endocrinology, Kasturba Medical College, Manipal, Manipal Academy of Higher Education, Manipal, 576104 Karnataka India; 5 Department of Cell and Molecular Biology, Manipal School of Life Sciences, Manipal Academy of Higher Education, Manipal, 576104 Karnataka India

**Keywords:** Deiodinase, Monocarboxylate transporter, Placenta, Pregnancy, *SLC16A2*, Thyroid hormone

## Abstract

The review aims to summarize the available research focusing on the importance of monocarboxylate transporter (*MCT8*) in thyroid hormone trafficking across the placenta and fetal development. A systematic search was carried out in PubMed; studies available in English related to “monocarboxylate transporter”, “adverse pregnancy”, “fetal development,” and “thyroid hormone” were identified and assessed. The references within the resulting articles were manually searched. *MCT8* is a highly active and selective thyroid hormone transporter that facilitates the cellular uptake of triiodothyronine (T3), thyroxine (T4), reverse triiodothyronine (rT3), and diiodothyronine (T2) in different tissues. *MCT8* is expressed in the placenta from the first trimester onwards, allowing the transport of thyroid hormone from mother to fetus. Mutations in *MCT8* cause an X-linked disorder known as Allan-Herndon-Dudley syndrome (AHDS), characterized by severe psychomotor impairment and peripheral thyrotoxicosis. Hence, any maternal thyroid dysfunction may cause severe consequences for the fetus and newborn. Further research regarding *MCT8* gene expression, polymorphic variation, and adverse pregnancy outcomes must be done to establish that *MCT8* is a novel prognostic marker for the early detection of pregnancy-related complications.

## Introduction

Thyroid hormones refer to 3,3′,5-triiodothyronine (T3) and 3,3′,5,5-tetraiodothyronine (T4) generated by thyroid gland follicular cells. The predominant bioactive thyroid hormone (TH) is T3, while T4 has very low intrinsic action [1]. THs are involved in forming several organs, including the brain, and regulating metabolic pathways and thermogenesis, throughout life [2, 3]. Moreover, T4 and T3 also have a prominent role in the development and metabolism of female reproductive organs such as the ovary, uterus, and placental tissues through particular nuclear receptors acting directly [4–7]. Untreated maternal thyroid disorders have been linked to pregnancy issues such as hyperemesis gravidarum, pre-eclampsia, stillbirth, miscarriage, and fetal growth restriction (FGR). This emphasizes the significance of maternal TH availability for proper fetoplacental development [8–10].

Although by mid-gestation, the fetal thyroid will be able to produce a substantial amount of T4, prior to that the fetus is dependent on the mother’s thyroid status. A steady flow of maternal THs through the placenta is essential for optimal fetal development [11]. Monocarboxylate transporter (*MCT8*) and other plasma membrane proteins capable of transporting THs enhance the maternal TH entrance into the trophoblast and its transfer across the placenta for fetal development [12].

*MCT8*, encoded by the solute carrier family 16 member 2 (*SLC16A2*), is a highly selective active transporter for TH and can be found in various organs, including the brain, where TH-sensitive neuronal populations are expressed. The presence of *MCT8* in choroid plexus and capillaries suggests its importance for TH transmission across the blood-cerebrospinal fluid barrier (BCSFB) and blood–brain barriers (BBB) [13]. Allan-Herndon-Dudley syndrome (AHDS), due to *MCT8* mutations, has a severe neurologic impairment and elevated serum T3 [14]. AHDS shows a thyroid profile that includes elevated free T3 (fT3), low reverse T3 (rT3), normal to low free T4 (fT4), and elevated or normal thyroid-stimulating hormone (TSH) in blood without any signs or symptoms of congenital hypothyroidism [15].

Scientific evidence reveals that *MCT8* gene expression in trophoblast is significant in the placenta’s cellular absorption of T4 and T3, not only for ideal placental function but also for fetal growth [16]. Since very few studies are available linking *MCT8* expression and adverse pregnancy outcomes, this review aims to summarize the existing evidence on the role of *MCT8* in maternal–fetal relations and intra-uterine fetal development.

### MCT Family

MCTs belong to the solute carrier 16 (*SLC16*) family of transporters and help transport short-chain monocarboxylates, hormones, minerals, and amino acids. There are 14 MCT isoforms (MCTs 1–14, *SLC16*A1-14) and two sodium-dependent MCT isoforms (SMCTs 1/2, SLC5A8/12) in the MCT family [17].

MCTs 1–4 (*SLC16A1*, *SLC16A7*, *SLC16A8*, and *SLC16A3*) were identified as true monocarboxylate transporters, whereas *MCT10* was discovered to be a T-type (aromatic) amino acid transporter, and *MCT8* (*SLC16A2*) is a TH transporter [1]. The proton-dependent transporters MCTs 1–4 help in the transfer of glycolysis products (lactate, pyruvate) and ketone bodies (acetoacetate, β-hydroxybutyrate) across the plasma membrane. *MCT1* could transport L-lactate into liver parenchymal cells and kidney proximal convoluted tubule cells for gluconeogenesis, where it is a crucial substrate, particularly after physical activity [18]. When compared to *MCT1*, *MCT2* shows a stronger affinity towards pyruvate and lactate [19] and is typically expressed in tissues that take in large amounts of lactic acid for use as a respiratory fuel (like neurons) or even for gluconeogenesis [18], whereas *MCT3* expresses and affects the lactate transport in the retinal pigment epithelium (RPE) [20]. *MCT4* is found in various tissues, but is particularly abundant in those that depend on glycolysis, like white skeletal muscle fibers, chondrocytes, astrocytes, several mammalian cell lines, and white blood cells [18].

Although recent research has revealed some knowledge on the substrate specificities of *MCT5*, *MCT6* has been demonstrated to transport drugs such as probenecid, nateglinide, and bumetanide. The remaining MCTs 5–14 have been less explored [17]. MCTs 5–7, 9, and 11–14 come under the category of orphan transporters with unknown substrates. *SLC16A10* was renamed *TAT1* instead of *MCT10* as it represents the T-type aromatic amino acid transporter [21]. Details of MCT isoforms are shown in Table 1. MCT tissue distribution in humans is listed based on “The human protein atlas database” (https://www.proteinatlas.org/) [22–32].

**Table 1:** MCT isoform tissue distribution in humans (NA; not available)

Protein name (MCT)	Uni gene name	Human gene locus	Predominant substrates	Transport mechanism	Tissue distribution	Accessory protein	References
*MCT1*	*SLC16A1*	1p13.2	Ketone bodies, lactate and pyruvate	H + cotransporter	Ubiquitous	CD147	22, 23, 32
*MCT2*	*SLC16A7*	12q14.1	Lactate, pyruvate and ketone bodies	H + cotransporter	Heart muscle, kidney, stomach, pancreas, heart, brain	EMBIGIN	22, 23, 24, 25, 26, 32
*MCT3*	*SLC16A8*	22q13.1	Lactate	H + cotransporter	Brain, retina, prostate, pituitary gland	CD147	22, 23,27, 32
*MCT4*	*SLC16A3*	17q25.3	Lactate, pyruvate and ketone bodies	H + cotransporter	Skeletal muscle, spleen, lungs, kidney, thyroid gland	CD147	22, 23, 28, 29, 32
*MCT5*	*SLC16A4*	1p13.3	NA	Orphan	Kidney, placenta, ovary, liver, choroid plexus	NA	22, 23, 32
*MCT6*	*SLC16A5*	17q25.1	Bumetanide, nateglinide	Facilitated diffusion	Kidney, stomach, small intestine, lungs, prostate, fallopian tube	NA	22, 23,30, 32
*MCT7*	*SLC16A6*	17q24.2	Pyruvate, lactate, ketone bodies	Orphan	Choroid plexus, retina, breast, spleen	NA	22, 23, 32
*MCT8*	*SLC16A2*	Xq13.2	T3, T4	Orphan	Liver, endocrine tissues, brain, female reproductive tissues	NA	16, 22, 23, 32
*MCT9*	*SLC16A9*	10q21.2	NA	Orphan	Kidney, adrenal gland, spleen, ovary, choroid plexus	NA	22, 23, 32
*MCT10*	*SLC16A10*	6q21	Aromatic amino acids (W, F, Y, L-DOPA)	Facilitated diffusion/exchanger	Choroid plexus, skeletal muscle, pancreas, placenta, liver, kidney	NA	22, 23, 31, 32
*MCT11*	*SLC16A11*	17p13.1	NA	Orphan	Thyroid gland, liver, skin, choroid plexus, kidney	NA	22, 23, 32
*MCT12*	*SLC16A12*	10q23.31	NA	Orphan	Kidney, choroid plexus, pancreas, placenta	NA	22, 23, 32
*MCT13*	*SLC16A13*	17p13.1	NA	Orphan	Liver, kidney, small intestine	NA	22, 23, 32
*MCT14*	*SLC16A14*	2q36.3	NA	Orphan	Salivary gland, brain, endocrine tissues	NA	22, 23, 32

MCTs have similar amino acid identities, projected topologies, and homology. There are 12 transmembrane (TM) domains in all MCT isoforms, as well as intracellular C and N termini and a large intracellular loop between TM 6 and 7 [33]. MCTs also rely on a range of auxiliary proteins for appropriate trafficking and activity at the plasma membrane. CD147 accessory protein is required for the co-expression of numerous MCTs, such as *MCT1*, *MCT3*, and *MCT4* [21]. Energy coupling (via H + or Na + cotransport), membrane insertion, and proper structural preservation depends more on the *N*-terminal domains, while the C-terminal domains are more significant for substrate specificity determination [33]. The sequence resemblance between these isoforms in various species is influenced by the evolutionary relatedness of their host species.

To enter the cells, THs require transporter proteins located in cell membranes. Changes in TH plasma levels can negatively impact all organs and organ systems and adversely affect the reproductive system [34].

The placenta has been discovered to express solute carrier family members (LAT1, LAT2, *MCT8*, and *MCT10*) and solute carrier organic anion transporter family members (OATP1A2, OATP4A1). These proteins appear to play a role in maternal–fetal TH exchange during the first trimester and trophoblast activity control (equilibrium apoptosis/cellular proliferation). *OATP1A2*, *LAT1*, *MCT10*, and *MCT8* mRNA expressions were significantly lower before 14 weeks in relation to full term. Although *OATP4A1* mRNA levels were comparable to pregnancy at 6–10 weeks, it peaked at the end of the first and beginning of the second trimesters [12]. *MCT8*, *OATP4A1*, and *LAT1* preferentially localize in the apical membrane of syncytiotrophoblasts (ST), which implies that these transporters directly involve TH absorption through maternal blood. However, current research has found that only *MCT8* is specific to the transfer of THs [12, 35].

### MCT8

**Fig. 1:**
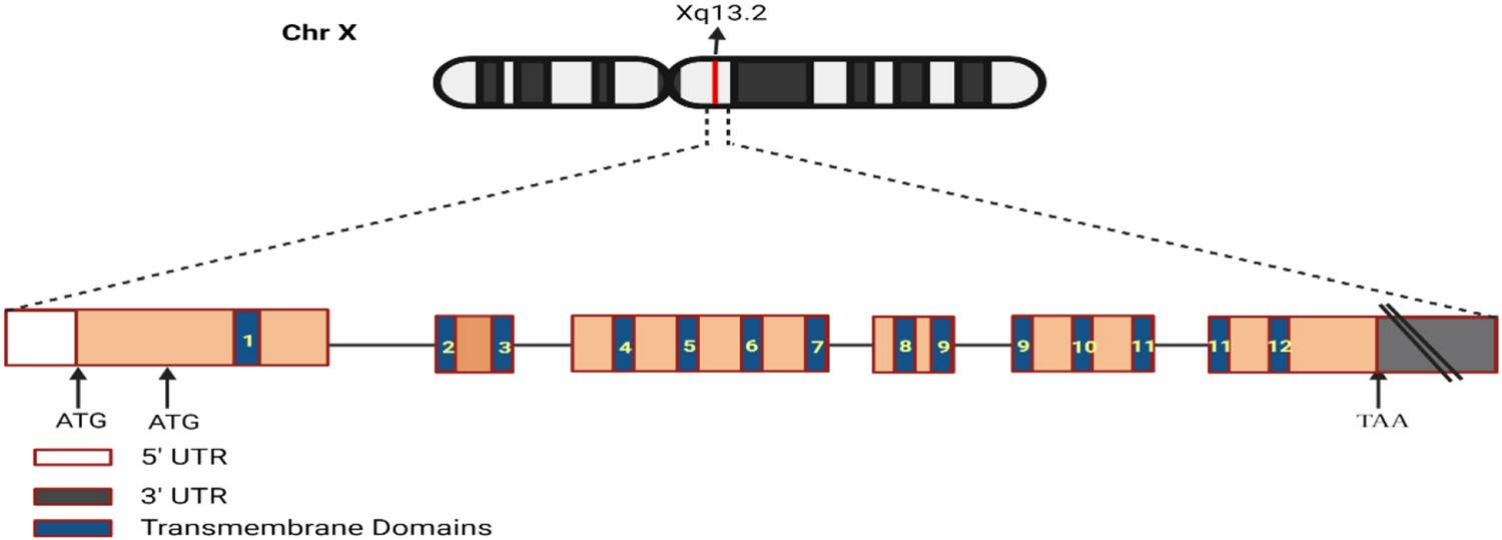
Genomic organization of *MCT8*/*SLC16A2*. Schematic representation of *MCT8* gene in X chromosome at position Xq13.2. ATG: transcription start site, TAA: transcription stop site

Monocarboxylate transporter 8 (*MCT8*), also known as *SLC16A2* or X-linked PEST-containing transporter, is a highly active and selective TH transporter that enhances the cellular absorption of triiodothyronine (T3), thyroxine (T4), reverse triiodothyronine (rT3), and diiodothyronine (T2). *MCT8* is a membrane transport protein that belongs to the monocarboxylate transporter (MCT) family of the major facilitator superfamily (MFS). *MCT8* and *MCT10* are the only members of the MCT family that transport iodothyronines [36]. It is positioned on the X chromosome, in the cytogenetic band of Xq13.2, and encodes a protein of 539 amino acids with a molecular mass of 59.5 kDa. *SLC16A2* has two in-frame translation start sites, which might code 613 or 539 amino acid proteins, respectively [1] (Fig. 1).

The *MCT8* gene covers approximately 112.6 kb of genomic DNA. The *MCT8* gene has six exons and five introns with an exceptionally long first intron. Depending on which of the two translation start sites is chosen, it generates either a 613 or 539 amino acid *MCT8* protein. *MCT8* contains 12 transmembrane domains (TMDs) and is found in both proteins, with the shorter one being substantially conserved in lower species. Exon 3 codes for four TMDs, while sections of two independent exons code for both TMD9 and TMD11 (TMD9 coded by exons 4 and 5, and TMD11 codes by exons 5 and 6); the 12 TMDs are spread among the six exons [36].

A) The physiological role of *MCT8*

**Fig. 2:**
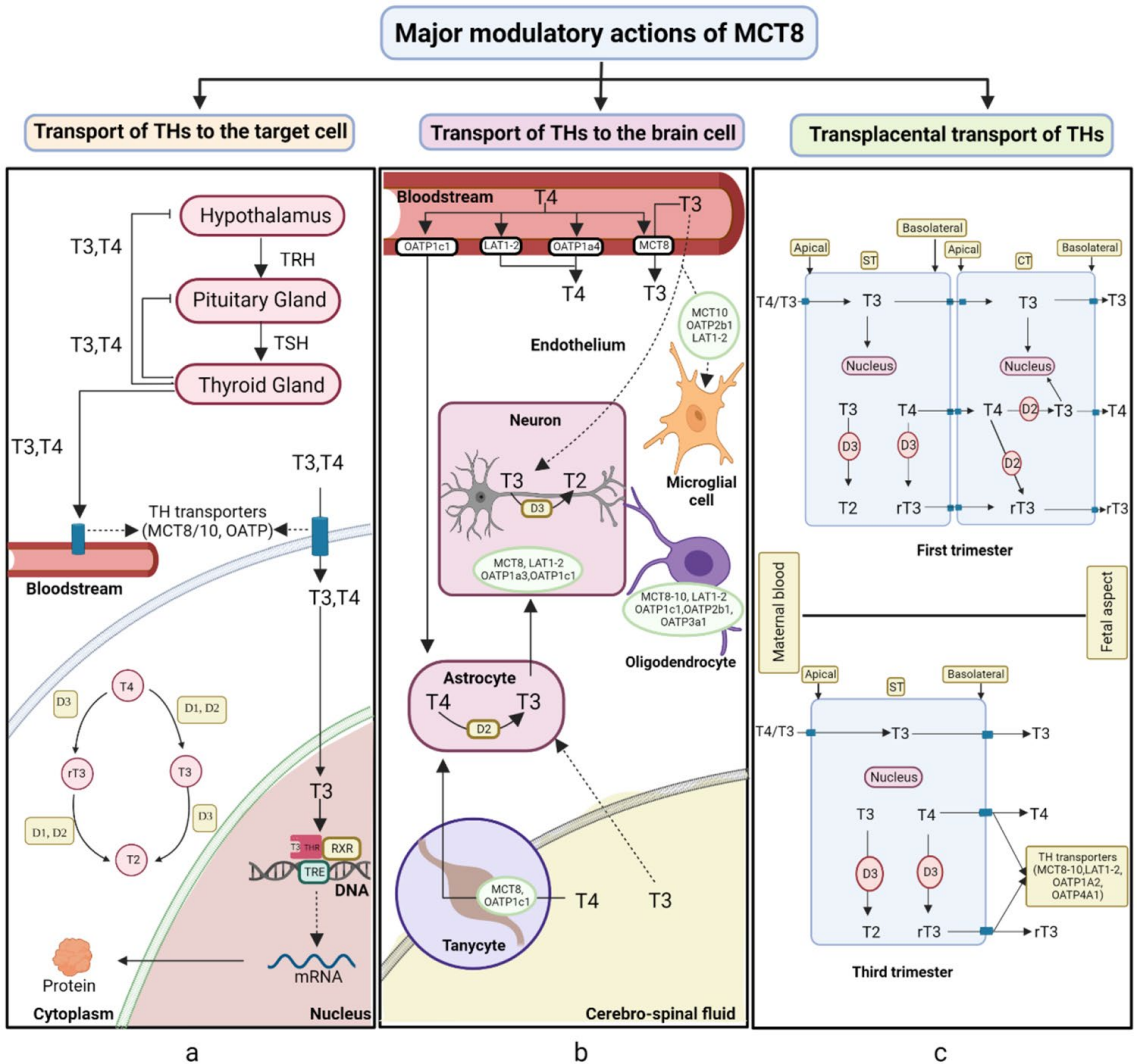
Major modulatory actions of *MCT8*. **a** The role of *MCT8* in transporting TH in blood and its action in the target cell. **b** The action of the *MCT8* transporter in TH uptake in brain cells. **c** The role of *MCT8* in the transplacental transport of TH. TRH: thyrotropin-releasing hormone, TSH: thyroid-stimulating hormone, THR: TH receptor, RXR: retinoid X receptors, TRE: TH response element CT: cytotrophoblast, ST: syncytiotrophoblast

*MCT8* is a TH transporter with a distinct function. TH, including the prohormone T4 and the active hormone T3, is essential for the formation of nearly all tissues and the basal metabolism, regulation, and regeneration of tissue [37]. In TH target cells, the plasma membrane transporter proteins enhance the cellular absorption and/or efflux of T4 and T3; moreover, deiodination enzyme 3 (DIO1-3) either activates or deactivates the TH and influences the T3 intracellular concentration [38, 39]. Only about 0.1% of the total circulating T3 or T4 will be in the unbound stage and will be able to reach cells via a specialized carrier-mediated pathway [40] (Fig. 2a).

Moreover, *MCT8* is vital for the T3 uptake into neurons, which plays a significant role in optimal neuron growth [41]. *MCT8* is found in the choroid plexus and the membranes of neuronal cells. Concerning the function of *MCT8* in the neuronal supply of T3, functioning astrocytes and neuronal units in the brain tissue regulate local T3 levels. Thus, local synthesis is the only source for the brain pool of T3, and once generated, neural cells can access T3 by a variety of membrane transporters [1]. This involves several processes, such as the uptake of T4 by astrocytes and the conversion of T4 to T3 by deiodinating enzyme 2 (D2). Later, this T3 will be released from the astrocytes, and *MCT8* regulates the neuronal uptake of T3. Finally, T3 will be transported via nuclear receptor to the neurons, and the subsequent breakdown of T3 by D3 will ultimately proceed in neurons (Fig. 2b) [42].

Mutations in this transporter have been linked to altered circulating T3 levels and neurological problems [43]. THs play a significant role in myelination, an important process in brain development [44]. Myelination delays have been reported in people with the MCT8 mutation impacting TH action on oligodendrocytes [45]. Many persistent myelination flaws in later life stages along with an increased proportion of small-caliber axons than the larger-caliber ones and defective oligodendroglial formation have been attributed to *MCT8* mutations. In spite of all of the above findings, the fundamental process of how *MCT8* mutations predispose to the above problems needs to be better understood [46].

The hypothalamus, a significant location for integrating TH feedback and gene control, expresses *MCT8* [45]. Families with an X-linked mental retardation condition known as the AHDS, first identified in 1944, also have mutations in the *MCT8* gene [47]. During gestation, the mother’s thyroid undergoes various alterations in response to the necessity of giving the fetus THs, until the fetal hypothalamus pituitary thyroid (H-P-T) system is completely functional. Clinical and subclinical hypothyroidism affects approximately 0.3% of women of reproductive age and 4.3% of pregnant women [48]. The bioavailability of TSH and the two physiologically active THs, T4 and T3, throughout the fetal period is influenced by factors such as (i) peripheral conversion of T4 to active T3 or to inactive metabolites and (ii) TH absorption and activation of cellular activities through binding to TH receptors [48]. Since THs are necessary for a fetus’s steady development and growth, even modest changes in the mother’s thyroid level during early gestation have been linked to neurodevelopmental complications in children later in life [49]. The deficiency or inadequate supply of THs during development might delay important processes like synaptogenesis, cell migration, neurogenesis, and myelination [50].

One of the other major modulatory roles of *MCT8* is in the transplacental transport of TH. The placenta aids in the regulation of maternal hormone transmission to the fetus. Deiodinases in the human placenta quickly convert maternal T4 to T3 to be used by the fetus, although a substantial portion of T4 is still transmitted to the fetus. TSH does not pass through the placenta as easily as iodide and thyrotropin-releasing hormone (TRH). The maternal TRH given to the fetus is thought to play a vital function in fetal thyroid function regulation before the H-P–T system matures completely [51]. *MCT8* is expressed in the human placenta as early as 6 weeks, with greater expression as the pregnancy progresses [12]. *MCT8* protein is found in the human placenta’s villous cytotrophoblast (CT), extravillous trophoblast, and ST [16]. Over the first trimester, the synchronized expression of *MCT8*, D2 (converting prohormone T4 to active T3), and the undifferentiated villous CT (trophoblast stem cell line) present in the TH receptors shows that T3 plays a role in the onset placental development [52]. The human placenta contains *MCT8* protein levels, particularly in primary villous CT, which are considerably greater in pregnancies complicated by FGR compared to gestationally matched adequately grown controls [53].

As the maternal THs T4 and T3 should pass through the ST’s maternal facing apical membrane and fetal facing basolateral membrane, together with CTs’ plasma membrane, they are metabolized by D2 and D3 expressed on the apical surface of STs. The expression of TH transporters and deiodinases varies with gestational age to maintain an optimum maternal–fetal transplacental supply of T4 and T3. So, the increased expression of *MCT8* in the maternal placenta could be a compensatory mechanism that helps trophoblasts to absorb T3 and transplacental TH transport (Fig. 2c) [54].

### MCT8 Gene Expression and Tissue Distribution

The liver and adrenal gland have the highest levels of *MCT8* mRNA, while the thyroid, brain, placenta, and kidney have slightly lower levels [1]. *MCT8* is localized in neurons and astrocytes of the paraventricular and infundibular nuclei in the human hypothalamus, according to Alkemade et al. [55]. Human tanycytes (ependymal cell type) line the third ventricle and are engaged in downregulation within the H-P–T axis, also expressed *MCT8* [23]. *MCT8* has been discovered in the arcuate nuclei of the hypothalamus, which play a significant function in TH-induced negative feedback control of TRH expression in humans [21].

*MCT8* is highly selective for the bidirectional transfer of T4, T3, rT3, and T2 across cellular membranes in mammals. However, its function in regulating T3 transport across the BBB is likely the most significant evidence that *MCT8* is involved in target tissue responses to circulating THs [56]. Strong immunoreactivity was found in all brain regions’ vascular structures, including in the surrounding astrocytes at GW32 and GW38, confirming the current assumption that *MCT8* is necessary for TH trafficking across the BBB. *MCT8* also showed high immunoreactivity in the subarachnoid space’s leptomeningeal cells and blood vessels at all ages. As a result, *MCT8* is found in both the inner and outer cerebrospinal fluid-brain barrier (CSFBB). Radial glial cells (RG), Cajal-Retzius cells, and cortical plate neurons all showed *MCT8* protein along their whole length [23]. D3, *MCT8*, and TR were detected in neurons in the paraventricular nucleus (PVN) that release TRH whereas D2 was discovered only in glial cells [36]. The neurological abnormalities are most likely due to a reduction in T3 absorption in *MCT8*-expressing central neurons, resulting in delayed brain development.

### MCT8 in Fetal Development

**Fig. 3:**
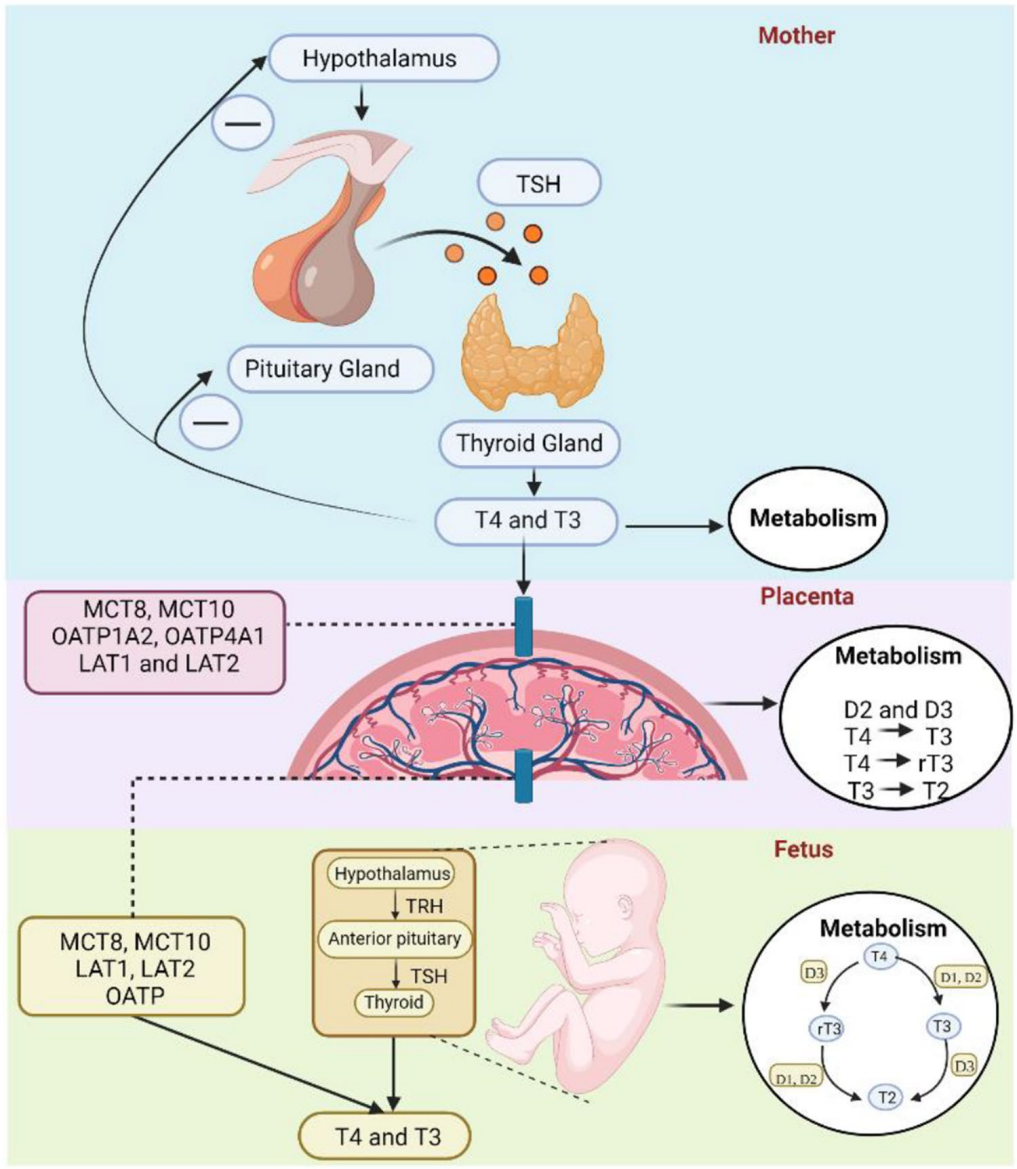
Bioavailability and metabolism of thyroid hormones in mother, placenta, and fetus

As the fetus grows, the amount of TH produced by the fetal gland rises while that produced by the mother’s gland declines (Fig. 3). Studies have shown premature hypothyroxinemia may have clinical implications such as an increased chance of cerebral palsy and increased risk of neurological and behavioral developmental issues [50].

Congenital hypothyroidism, maternal hypothyroxinemia and hypothyroidism, mutations in T3 receptors, and the gene encoding the TH-specific transporter *MCT8* are all examples of causing adverse conditions in pregnancy [57].

TH transporters and deiodinases control the amount of TH available to brain cells. Transporters and integral membrane proteins mediate the cellular inflow and efflux of TH. TH requires transporters to traverse the BBB [38] and, to a lesser extent, the BCSFB, according to studies in postnatal mice. Two additional barriers are present in the fetal brain: the outer and inner CSFBB. The external CSFBB, which is made of intercellular connections among pial leptomeningeal cells and the basal end-feet of RG, prevents molecules from passing from the subarachnoid space in the cerebrospinal fluid (CSF) into the cerebral cortex [58]. Strap junctions produce the inner CSFBB in the neuroepithelial cells that line the ventricular system and evolve into RG, which fades later in development. The interchange of chemicals between the CSF and the ventricular zone (VZ) is restricted by this barrier. At this time, it is uncertain whether these obstacles influence the availability of TH in the brain [59, 60]. *MCT8* deficiency limits TH entry into the brain; T4 and T3 levels have been measured in the brain of an *MCT8* defective human fetus, though in lower proportions. This is most likely mediated by an alternative transporter known as OATP1C1 [58], found in the human embryonic brain. As a result, it seemed rational to expect that boosting T4 levels in the blood would increase the quantity of TH available to the brain. T4 was chosen as the preferable TH for two reasons: first, fetal blood T3 does not accumulate in the brain; second, intra-amniotic T4 rather than T3 has been employed in treating fetal goiter [61, 62]. T4 and T3, as well as other TH derivatives, are transported via *MCT8* [63]. T4 and steroid hormone metabolites are transported by OATP1C1, while T3 is not [64].

López-Espndola et al.’s study demonstrated *MCT8*’s existence in the human fetal BBB and in leptomeningeal cells by immunohistochemistry and in situ hybridization. This association of *MCT8* with microvessels and the astrocytes helps in transport of T4 across the BBB and its deiodination to T3. The study also found that the *MCT8* was highly expressed in the BBB, but DIO2, DIO3, and *OATP1C1* were barely expressed. This implies the significance of *MCT8* in transferring TH to the brain throughout development [58].

*MCT8* and *OATP1C1* immunoreactivity has been detected since GW14 [14]. According to cortical T3 concentration, from GW12 to GW14, DIO2 activity increases in the cortex, but DIO3 activity decreases. T3 receptor protein levels in the fetal brain rise to 2000 molecules per nucleus between GW10 and GW18, as evaluated by T3 binding. While important T4 and T3 transport occurs across the BBB, ubiquitous *SLC16A2* expression suggests that *MCT8* may also play a role in neuronal T3 transport. During the second trimester, fetal thyroid production, cortical DIO2 activity, T3 receptors, and concentration of T3 all grow consistently in the presence of a stable maternal T4 supply [65].

The presence of *MCT8*, *OATP1C1*, DIO2, and DIO3 in migratory streams in the hippocampus and brainstem, as well as the subiculum and presubiculum cells, indicates that these proteins modulate local TH concentrations in these locations to control cell migration [58]. According to the findings, *OATP1C1* has a primary role in the entrance of circulating TH to the human fetal brain due to its low expression in the prenatal human BBB, in comparison to what is observed in rodents, and its abundant expression in choroid plexus epithelial cells, ependymal cells, tanycytes, and leptomeningeal cells [66]. This could explain why *MCT8* deficiency causes delayed cerebral cortex and cerebellar maturation, abnormal synaptogenesis, and hypo-myelination in humans during prenatal stages [67].

A) Pathophysiology of *MCT8* deficiency and Allan-Herndon-Dudley syndrome

In patients with *SLC16A2* gene mutation, the importance of proper *MCT8*-mediated TH transport becomes evident. Such mutations can characterize a phenotype of severe intellectual and motor impairment and indications of peripheral thyrotoxicosis. Since *SLC16A2* is on the X chromosome, it predominantly affects men [68]. Pathogenic mutations in the *SLC16A2* gene cause AHDS, an X-linked recessive disorder [69]. Males with *MCT8* mutations or AHDS have both hyper and hypothyroid tissues. *MCT8* mutation carriers in females do not have any AHDS symptoms [36]. Mental and developmental motor delays characterize AHDS and thyroid functioning abnormalities such as high blood T3, low T4, and normal or mildly elevated TSH [70]. According to a multicenter cohort study, 30% of AHDS patients died in childhood, with pulmonary infection (18.8%), aspiration pneumonia (9.4%), and sudden death (18.8%) being the leading reasons for death [71]. Over 100 mutations in *SLC16A2* have been linked to AHDS thus far. Most clinically significant missense mutations affect residues predicted to be found within TMDs. Some mutations are thought to disrupt substrate translocation (e.g., p.R445C and p.D498N), while others impact protein trafficking and stability (e.g., p.G282C and p.G558D) [47].

The residual transport ability of the mutant *MCT8* protein has been connected to the severity of the clinical phenotype. Severe abnormalities have been associated with frameshift mutations and substantial deletions and are often connected with a shorter life expectancy and poor quality of life [72]. Necropsies of brain tissue from *MCT8*-deficient patients have demonstrated brain changes consistent with hypothyroidism such as abnormalities of neuronal differentiation, myelination, and synaptogenesis that have been evident even during the prenatal period [73].

According to the study conducted by Ramos et al., *MCT8* mutation detected during pregnancy can help in the early detection of AHDS in newborns [74]. The research work done by Vatine et al. studied induced pluripotent stem cell (iPSC)-derived neural cells lacking *MCT8* along with brain microvascular endothelial cells derived from iPSC (to mimic the diseased *MCT8*-deficient human BBB) to evaluate the potential involvement of BBB in TH absorption and showed normal T3-dependent neuronal development in spite of reduced TH absorption. This showed that *MCT8* is necessary for T3 transport across the BBB, which is probably the cause for the lower T3 concentrations seen in *MCT8*-deficient brains [75]. As a result, a BBB created utilizing stem cells from AHDS patients could not transfer TH properly [75, 76]. These results imply that decreased TH transport across the BBB may be a major factor in the abnormal neurologic phenotype of AHDS [77].

Refetoff et al. demonstrated prenatal therapy for *MCT8* deficiency using L-T4 and TH analogs. They showed that a large dose of intraamniotic administration of L-T4 resulted in increased T3 production and decreased TSH in the fetus (as measured in the amniotic fluid) [78]. The TH analogs diiodothyropropionic acid (DITPA), Tetrac, and Triac, thyromimetics can pass the placenta without the need for *MCT8* transporter activation and hold promise for therapeutic use in such cases [79]. Preclinical results show that Triac administered at birth entirely prevented aberrant brain development in animal models of *MCT8* deficiency [80].

B) *MCT8* polymorphism

In addition to mutations, such as those found in AHDS, other genetic variations, such as genetic polymorphisms, also contribute to inter-individual diversity [81]. Changes in the human genome’s nucleotide sequence, known as polymorphisms, affect at least 1% of the general population. In addition to determining individual characteristics like hair or eye color, these polymorphisms also contribute to the variance in serum TH levels between people [82]. rs6647476 and rs5937843 were the two major *MCT8* polymorphisms studied so far.

Serine-to-proline substitution at position 107 (Ser107Pro; rs6647476) *MCT8* polymorphism was the topic of investigation by Lago-Leston et al. [83]. They discovered no correlation between TH-responsive genes in T3-stimulated fibroblasts or in, white blood cells, or *MCT8* coded by mRNA levels or with serum TH levels [83]. A study conducted by van der Deure et al. identified that hemizygous carriers of rs5937843 polymorphism found in the *MCT8* gene’s intron 5 exhibited lower fT4 levels in comparison to male participants who were wild-type [84].

A study by Roef et al. shows that the two *MCT8* SNPs were related to circulating TH levels in men but not women. rs5937843 was inversely associated with fT4 concentrations in men but not in women, and rs6647476 was negatively associated with fT3 levels in men. They concluded that these two *MCT8* SNPs were associated with male TH levels but not females [85]. These results suggest that common genetic variations in the *MCT8* gene, depicted in Table 2, may impact male TH levels.

**Table 2:** Common variations present in *MCT8* and its impact on TH levels

Transporter	rs-number	Location	Gene	Change	Impact on TH levels	References
*MCT8*	rs5937843	Intron 5	Xq13.2	T > G	Hemizygous male carriers; • Positive correlation with TSH • Lower fT4 concentrations • Inverse relations with total T4 and rT3 levels	85
	rs6647476	Exon1-missense	Xq13.2	Ser107Pro (T > A, C)	Hemizygous male carriers; • Lower levels of fT3	85

### MCT8 Expression and Adverse Pregnancy

The study by Chan et al. observed an increase in *MCT8* mRNA levels in the human villous placenta afflicted by severe FGR necessitating preterm delivery compared to the normal placenta. Increased *MCT8* expression could be another compensatory mechanism in the fetoplacental unit, seeking to improve T3 absorption in trophoblast cells or TH transplacental passage in these adverse pregnancies [16].

Increased *MCT8* protein expression will likely contribute to the enhanced net T3 uptake seen in FGR CT. Additionally, the high T3 intracellular binding to TR isoforms (TRα1 and TRβ1) expressed at placenta villi may also contribute to the increased accumulation of T3 within CT of the FGR. This might lead to an increase in the intracellular accumulation of T3 and an increase in the FGR CT’s sensitivity to T3 [53].

Chan et al.’s study showed increased *MCT8* expression in the FGR human placenta as compared to the fetal cerebral cortex. They showed a decreased *MCT8* expression in the FGR fetal CNS with increasing growth restriction. In conclusion, decreased *MCT8* expression in the FGR fetal CNS may contribute to long-term neurodevelopmental abnormalities [67].Compared to spontaneous preterm birth, infants with an indicated preterm birth group showed greater circulating T3 levels. Irrespective of the cause of preterm birth, preterm infants had hypothyroxinemia. However, maternal and placental compensatory responses such as TSH, *MCT10*, D2, and D3 were present only in indicated preterm birth but not in spontaneous preterm birth. When comparing spontaneous and indicated preterm birth, the group of indicated preterm births had significantly higher *MCT10* protein levels. Between spontaneous/indicated preterm birth and normal pregnancies, the *MCT8* mRNA and protein levels were the same [86].

Any degree of glucose intolerance that begins during pregnancy is considered gestational diabetes mellitus (GDM). For the fetus to develop normally during pregnancy, there must be an appropriate concentration of THs. In normal pregnancies, the steady concentration THs fT4 and T3 carried through the placenta from the maternal blood to fetus is maintained by D2 and D3. However, the expression and activity of D3 and D2 are affected in GDM, resulting in less T3 transfer to the fetal circulation. GDM women had greater TH levels than normal mothers, with both Total T3 (TT3) and TSH levels peaking in the third trimester. On the other hand, the newborn had lower fT4 and TT3 in the umbilical cord blood. *MCT8* and OATP-E expression is lower in trophoblast cells from GDM pregnancies [87].

Nijkamp et al. showed placental hypoplasia, and fetal hydrops could be two underlying mechanisms that contribute to intrauterine fetal death (IUD) that are associated with thyroid dysfunction [88]. Although studies link thyroid dysfunction with IUDs, there is no clear proof of how *MCT8* expression affects IUDs.

## Conclusion

Thyroid dysfunction has been linked to various human behavioral, physiological, and morphological abnormalities, including reproductive and developmental issues. Identification of *MCT8* gene expression associated with various pregnancy outcomes might help to comprehend the developmental behavior of fetal life. *MCT8* as a predictive marker may allow for early stratification and intervention in women at high risk of developing an unfavorable pregnancy outcome. Thus, evaluating *MCT8* gene expression levels in correlation to various pregnancy outcomes could be a novel approach for validating the roles of this marker in detecting pregnancy-related complications in advance. Nevertheless, future preclinical and clinical studies are needed to examine *MCT8*’s polymorphic forms’  in relation to pregnancy outcomes.

## Data Availability

No datasets were generated or analyzed during the current study.
